# Assessing clarity and erasability of commercially available pens for surgical site marking: a comparative study in human volunteers

**DOI:** 10.1186/s13037-016-0097-6

**Published:** 2016-03-18

**Authors:** F. C. J. Sim, D. Angadi, G. E. Jarvis, M. Porteous

**Affiliations:** Trauma and Orthopaedics Department, West Suffolk Hospital, Hardwick Lane, Bury St Edmunds, Suffolk IP33 2QZ UK; Selwyn College, Cambridge University, Grange Rd, Cambridge, CB3 9DQ UK

## Abstract

**Background:**

Marking the surgical site is a well-established part of pre-operative protocol and errors in marking have been implicated in wrong site surgery incidents and are a significant patient safety issue. There are many commercially available marker pens and anecdotally very little consistency in which pen is used or the clarity of marking. Previous studies have shown subjective differences between different pens and the current paper sought to support this evidence with objective data and widen the investigation of commercially available pens.

**Methods:**

Eight marker pens were used to mark two separate sites on three caucasian volunteers. These marks were photographed and assessed by six observers before and after the application of chlorhexidine skin preparation. The observers were blinded to which pen was used for each mark, and rated the clarity of the marks subjectively. The photographs were assessed using image analysis software to give an objective measure of clarity against the skin.

**Results:**

There was a wide variation between the clarity of marks made by the different pens, and also a wide variation in the resistance to skin preparation. The Pentel N50 pen was the outstanding best performing pen across all categories.

**Conclusions:**

It is recommended that the Pentel N50 black marker pen be used for surgical site marking to improve patient safety and avoid adverse events.

## Background

Marking the correct surgical site clearly before surgery with an indelible marker pen is an established part of pre-operative protocol [1, 2]. Guidelines recommend that the mark should be visible in the operative field before making any incision in the patient. Breaches of this protocol have been implicated in wrong site surgery incidents [3].

Preparation of the surgical field using alcohol based solutions containing chlorhexidine or povidone-iodine is routine practice to minimise the risk of surgical site infection [4]. In some cases an additive dye is used to avoid the surgical team missing an area of skin when applying the skin prep solution. Most of the inks contained in indelible markers are alcohol soluble and can be partially or completely washed off during skin preparation [5–7], or if the patient has a bath or shower after they have been marked but before surgery.

Within our department a number of different commercially available indelible marker pens were historically used to mark surgical sites. The marker type seemed to be determined more by the special offers available to the hospital supplies department than any objective criteria. Furthermore there was considerable variability in the different markers’ resistance to fade following skin preparation.

For these reasons the current study was undertaken to compare the clarity of the marks of a variety of commercially available indelible marker pens in the United Kingdom.

## Methods

**Fig. 1:**
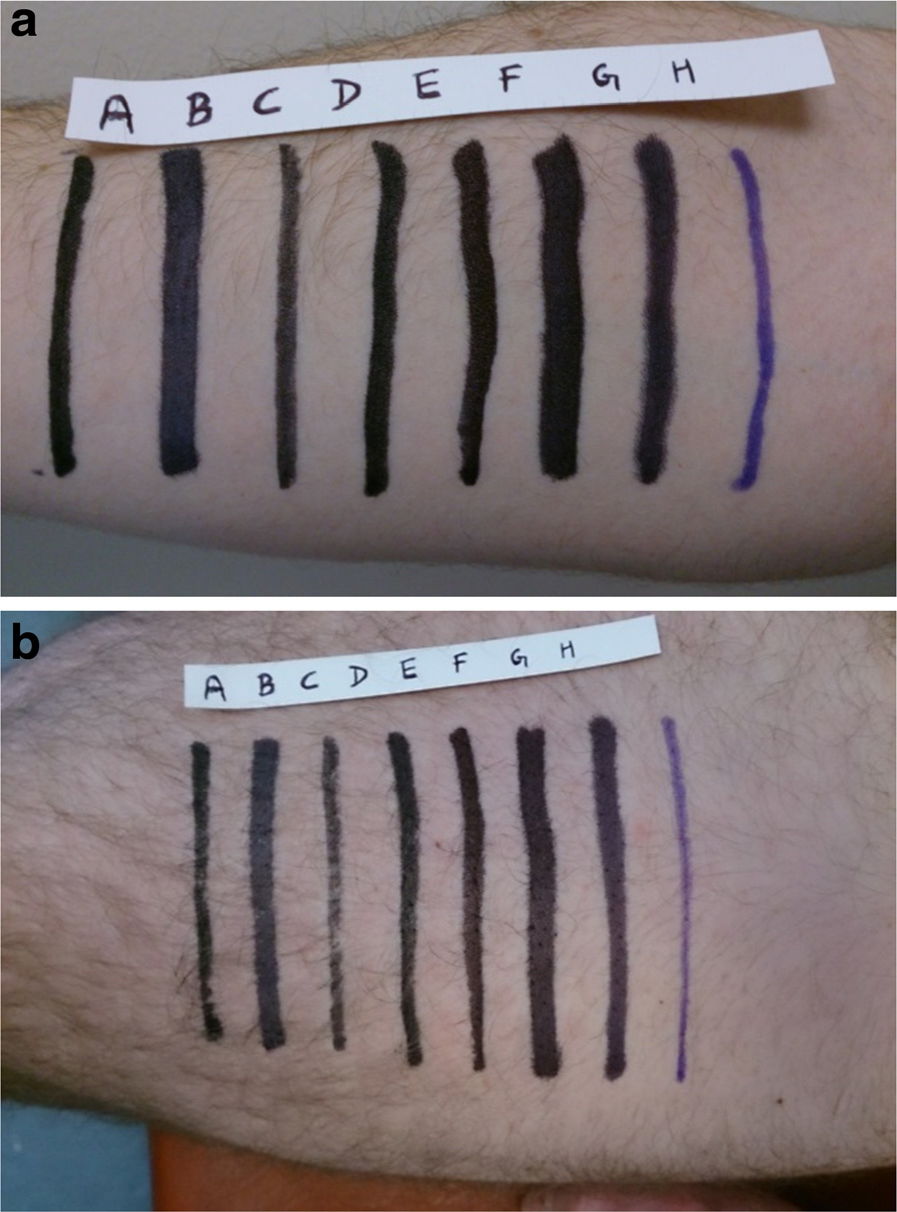
Examples of marking sites on both upper (**a**) and lower (**b**) limbs prior to chlorhexidine preparation. A - Edding 300, B - Niceday Chisel 1-5 mm, C - Rosinco Friendly bullet, D - Pentel N50, E - Staples Duramark, F - Sharpie W10, G - Foray Recycled and H – Viomedex

Eight commercially available and commonly used indelible marker pens were used to mark the skin of three volunteers. The markers used were (a) Edding 300, (b) Niceday Chisel 1–5 mm, (c) Rosinco Friendly bullet, (d) Pentel N50, (e) Staples Duramark, (f) Sharpie W10, (g) Foray Recycled and (h) Viomedex. Sites on the lateral aspect of the thigh and anterior aspect of the forearm were marked on each volunteer, giving six sites in total. Hairy skin was not shaved before marking as NICE guidelines suggest that this should routinely be done in the operating theatre [4]. Each pen was used to draw a mark with the broadest possible single stroke. As seen in Fig. 1a and b, the equidistant marks on the forearm and thigh were 5 cm and 7 cm long respectively.

After the ink had been allowed to dry, the skin was prepared with a solution of Chlorhexidine Gluconate 2 % w/v in Isopropanol 70 % v/v (Ecolab, Leeds, UK) with an added dye (Carmoisine E122 1.5 % w/v). Skin preparation consisted of two coats of solution applied with a surgical swab as per the manufacturer’s recommendations.

The six sites on the three volunteers were assessed by six observers. The observers comprised of members of the orthopaedic team including medical, nursing and theatre staff. Both the volunteers and the observers were blinded with regards to the type or trade name of the indelible marker pen. Each observer viewed the set of marks before application of the chlorhexidine solution. Following the skin preparation each observer graded on a proforma whether each mark was 0, 25, 50, 75 or 100 % as clear as the mark had been prior to any skin preparation. Each observer graded the mark individually and at the bedside to avoid their decisions being influenced by others.

**Fig. 2:**
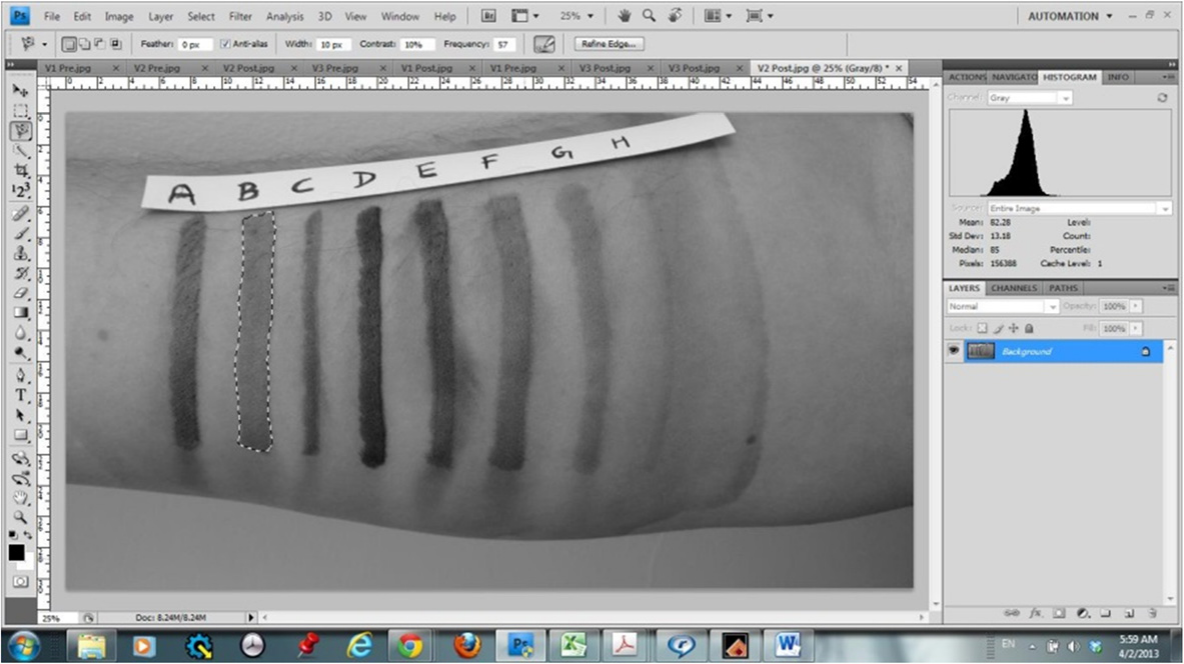
Demonstration of the process of obtaining the grayscale data using Adobe Photoshop CS4 (Adobe, San Jose, California, USA) histogram tool

Photographs of each set of marks were taken before and after skin preparation using a digital camera (Xperia™, Sony) having 28 mm equivalent fixed lens with fast aperture f/2.4 and 13 megapixel Exmor RS image sensor. Using Adobe Photoshop CS4 (Adobe, San Jose, California, USA) the raw digital images were converted into a grayscale with 256 levels (0 being the darkest and 255 being white). The histogram tool was used to assess the grayscale level of the individual marks in the pre and post skin preparation photographs (Fig. 2). The difference between the two grayscale levels represented the change in the contrast of each mark as a result of skin preparation.

## Results

Data was collected using two methods as described above: grayscale photo analysis and observer rated clarity. The results of the study are therefore presented in these two groups with a final section comparing the two sets of data.

### Grayscale data

**Fig. 3:**
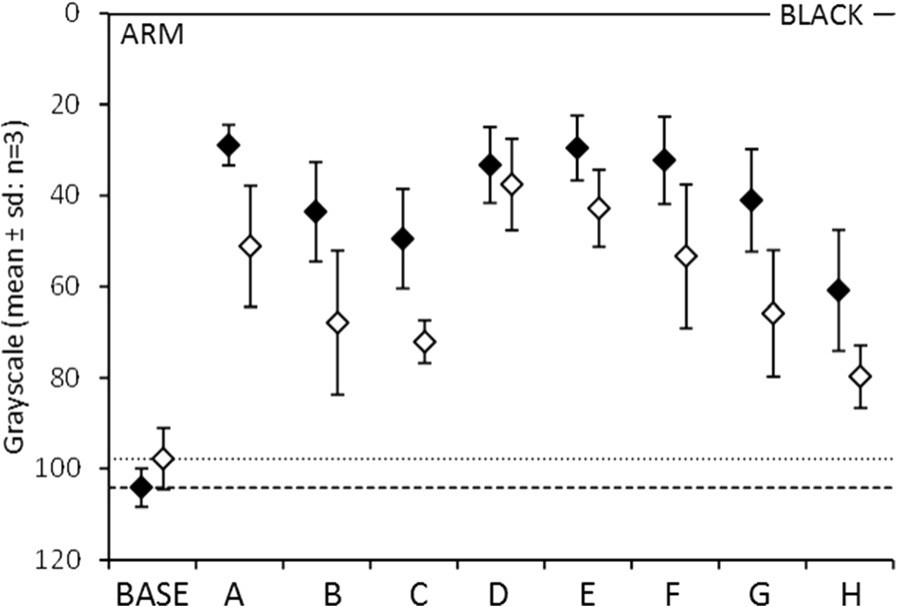
Scatter chart displaying the raw grayscale data for marks made on the upper limb. The *y-axis* represents the level of *grey* with 0 being *black*. Along the *x-axis* are represented the different markers and a base level representing bare skin. *Filled diamonds* show figures prior to surgical preparation and *unfilled* show figures after

**Fig. 4:**
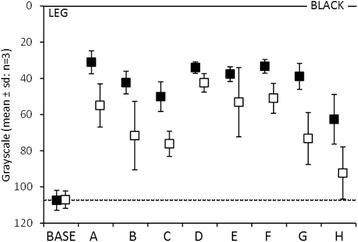
The raw grayscale data for marks made on the lower limb, represented in the same manner as in Fig. 3 for the upper limb

The raw data from the upper (Fig. 3) and lower (Fig. 4) limb markings demonstrate that each pen made an initial mark on the skin that was significantly different to bare skin. The clarity of this mark varied, with pens A, D, E and F the most clear and pen H the least.

After surgical preparation with Chlorhexidine solution each pen mark became less dark as compared to bare skin, however some marks were more susceptible to surgical preparation than others. Pens B, C, G and H all showed the least resistance to Chlorhexidine preparation, whereas pen D was the most resistant.

All of the pen marks displayed a difference to bare skin after surgical preparation was applied, however the difference between these marks and bare skin varied. Pen H performed the least well and pen D performed best with the greatest clarity following surgical preparation, as seen in Table 1.

**Table 1:** Represents the modelled grayscale data for marks made on both upper and lower limbs, the variability of the marks and the effect of surgical prep on each mark

Pen	Mark	Mark arm SD	Mark leg SD	Prep (+/−SD)
A	0.684	0.065	0.038	0.278 +/−0.082
B	0.595	0.057	0.033	0.417 +/−0.123
C	0.519	0.049	0.029	0.418 +/−0.124
D	0.649	0.062	0.036	0.005 +/−0.001
E	0.675	0.064	0.038	0.213+/−0.063
F	0.683	0.065	0.038	0.277 +/−0.082
G	0.620	0.059	0.034	0.421 +/−0.125
H	0.411	0.039	0.023	0.481 +/−0.142

The data model standardises the value for the initial mark (Mark) and the effect of skin preparation (Prep) so that they are the same for both the upper and lower limbs. In the column headed “Mark” a value of 0 indicates no discernible mark and a value of 1 indicates a completely black mark. In the column headed “Prep (±SD)” a value of 0 represents no effect of surgical preparation, and a value of 1 represents complete removal of the mark by surgical prep. The columns headed “Mark Arm SD” and Mark Leg SD” represent the variability of clarity of the marks made on upper and lower limbs

There was no statistical evidence of any difference between either the clarity of the pen mark nor the effect of surgical preparation between sites on the upper and lower limb. There does, however, appear to be a greater variability of the clarity of marks made on the upper limb as opposed to the lower limb. This is demonstrated in Table 1.

### Observer data

**Fig 5:**
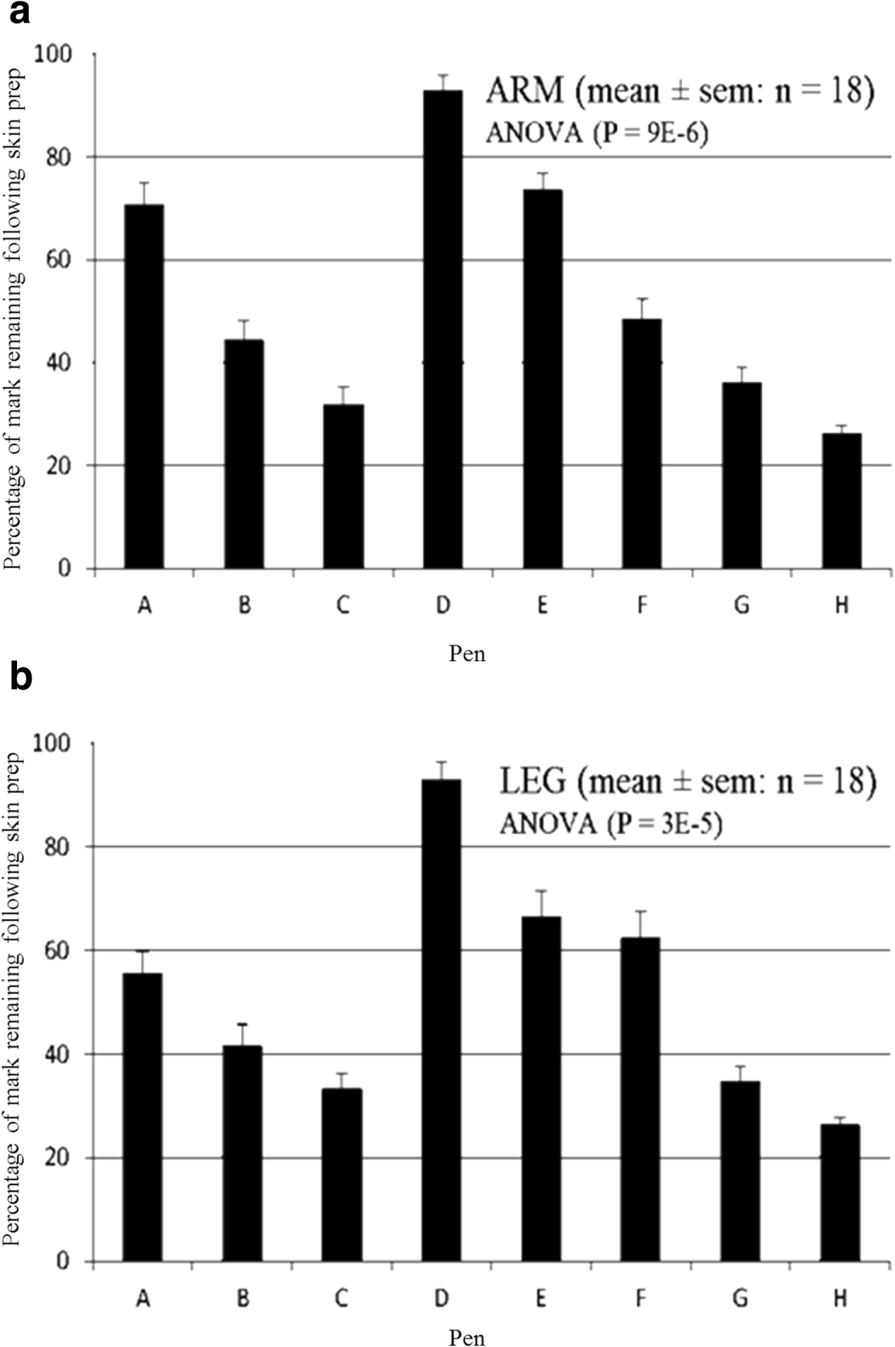
Graphs to demonstrate the change in percentage clarity for upper limb (**a**) and lower limb (**b**) sites for each pen. Data is taken from all six observers and all three volunteers

The observer data was analysed using univariate ANOVA modelling in SPSS (IBM SPSS Statistics 21.0, IBM Corp., Armonk, NY, USA) (Fig. 5a and b). This reveals a significant statistical difference between the clarity of the pens after Chlorhexidine preparation. Pen H is consistently the worst performing, and pen D the outstanding best performer, as further supported by post-hoc analysis using a Waller-Duncan test.

**Fig. 6:**
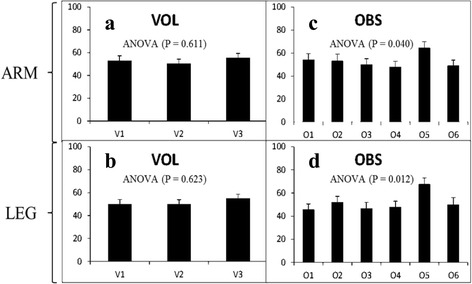
**a** and **b** Demonstrate the modelled data for each volunteer for upper limb and lower limb sites respectively. This encompasses data obtained for all pens viewed by all observers. **c** and **d** Demonstrate the modelled data for each observer for upper limb and lower limb sites respectively. This encompasses the data obtained for all pens across all volunteers

The ANOVA statistics also reveal that there is no significant difference in the percentage change in clarity following Chlorhexidine preparation between the volunteers for all pens, suggesting that the volunteer has no effect on the clarity of the mark in this setting (Fig. 6a and b). There was a difference between observers, however. Observer five consistently marked higher than the other five observers, suggesting that clarity may be in the eye of the beholder (Fig. 6c and d).

### Comparison of observer vs. grayscale data

**Fig 7:**
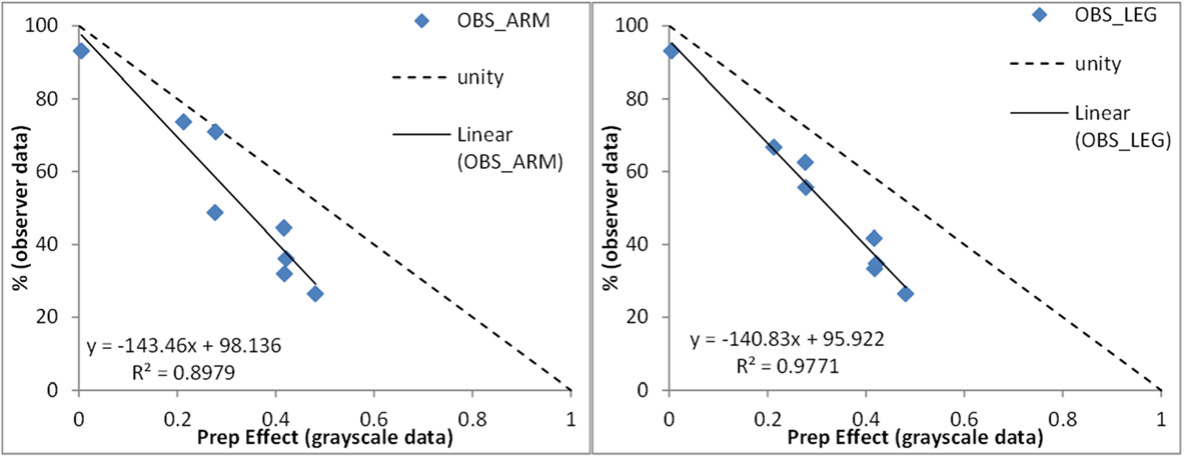
Demonstrate the relationship between the observer data and the grayscale data comparing the PREP parameter from the grayscale data (0–1) to the synonymous change in percentage clarity parameter from the observer data (1–100 %) for the upper and lower limb sites respectively. The unity line represents complete agreement between the two sets of data

Both observer and grayscale data support the final conclusions that Pen D is the best performing and pen H is the worst. Furthermore, when the two synonymous parameters (namely the change in clarity following Chlorhexidine preparation) from each data set are compared there appears to be a close relationship between the two indices (Fig. 7). There is, however, a tendency for the observers to overestimate the effect of the Chlorhexidine preparation as compared to the grayscale photo analysis.

## Discussion

Adverse events in surgery, in particular ‘wrong site surgery’, can lead to significant harm for patients [8, 9] and appropriate surgical site marking as part of the universal protocol for safe surgery published by the World Health Organisation [1], WHO, has an important role in reducing the risk of these events [10, 11]. The WHO protocol states that the surgical mark should be unambiguous, clearly visible and made with a permanent pen so that the mark is not removed during site preparation. The patient should also play a role in the placement of the mark, with the mark made before the patient arrives in theatre [12]. There is therefore a need for a pen which is resilient to the surgical preparation solutions. There is also growing evidence that alcohol-based solutions are the most effective in preventing surgical site infections [13], and it is these alcohol-based solutions that have the most deleterious effect on the clarity of surgical site markings.

In our study we noted that the clarity of the skin mark varied amongst the indelible pens routinely marketed as ‘permanent’. In addition to the chemical composition of the ink there are a few plausible factors for this observation; the width of the marker tip helps determine the relative size of the mark and the material composition of the marker tip also plays a role in the distribution of the ink on the skin.

We selected the six observers from different backgrounds in a multi-disciplinary team to reflect an operative team which routinely performs the preoperative surgical checklist [14]. Our results show that all observers found the same pen to be the highest performing, and the same to be the lowest performing reflecting a low inter-observer variability. The lowest performing pen was also the only pen studied to be marketed specifically as a surgical site marker and the most expensive per unit.

Currently there is a lack of standardisation on the size of the mark that needs to be made. The findings here show that there was no significant difference in the average clarity of marks made on the arm (5 cm) as compared to the leg (7 cm). There was however greater variability in the clarity of the smaller mark on the arm. This suggests that there is less consistency in the clarity of smaller marks and that making a larger mark where possible is likely to result in the clearest mark.

Other considerations in surgical site marking should include the theoretical risk of cross-contamination between patients, particularly in immunocompromised or MRSA colonised patients where a single use pen may be appropriate [15]. The mark should also be placed in a position that will be visible in the surgical field [12]. The manufacturer safety information provided with some of the permanent markers suggests that they can have an irritant effect when brought into contact with skin [16], which should be monitored for each patient.

The authors acknowledge that the study has limitations; although there is no significant variation in the mark across the volunteers, all three volunteers were Caucasian and therefore variation the clarity of skin marking on other skin types cannot be commented on. Also, the recollection of the observer was relied upon in order to estimate the effect of skin preparation on the mark. This was intentional in order to simulate the operative environment and also to ensure that the observers made an individual assessment of the mark, without influence from the other observers. It did however mean that the observer was not able to make a direct comparison with a fresh mark, and a prepared mark simultaneously.

Finally, it should also be mentioned that Pen H was of a different colour to the other pens. It was included in the study as it is marketed specifically as a surgical site marker and therefore should be compared in this context, regardless of colour. The methods of analysis in both the observer and grayscale data collections were also designed to compare the contrast of the mark to the surrounding skin, rather than the colour.

## Conclusions

A large (7 cm) mark with the Pentel N50 Black indelible marker, regardless of surgical site, gives the clearest mark of those pens tested and therefore should be used to reduce the risk of wrong site surgery and improve the confidence of all theatre staff and patients in the safe marking of the surgical site.
